# Association between the APC gene D1822V variant and the genetic susceptibility of colorectal cancer

**DOI:** 10.3892/ol.2014.2102

**Published:** 2014-04-28

**Authors:** MAOHUI FENG, XIPING FANG, QIAN YANG, GANG OUYANG, DAPING CHEN, XIANG MA, HUACHI LI, WEI XIE

**Affiliations:** 1 Department of Oncology, Zhongnan Hospital of Wuhan University, Wuhan, Hubei 430071, P.R. China; 2 Department of Oncology, The Central Hospital of Enshi Prefecture, Enshi, Hubei 445000, P.R. China; 3 Key Laboratory of Aquatic Biodiversity and Conservation, Institute of Hydrobiology, Chinese Academy of Sciences, Wuhan, Hubei 430072, P.R. China

**Keywords:** colorectal cancer, APC gene, single nucleotide polymorphism, genetic susceptibility

## Abstract

Adenomatous polyposis coli (APC) gene polymorphisms are believed to contribute to tumor susceptibility. However, the association between genetic variants (A/T) in the APC gene D1822V polymorphism and colorectal cancer (CRC) susceptibility remains unknown. To determine this association, a case-control study was performed. The genotype of the APC gene D1822V variants was analyzed by DNA sequencing in blood samples collected from 196 patients with CRC and 279 healthy subjects. There were no significant associations between the case and control groups in the distribution of AT [odds ratio (OR), 0.604; 95% confidence interval (CI), 0.355–1.029) and TT genotypes (OR, 0.438; 95% CI, 0.045–4.247) relative to the AA genotype. The ratio of the T allele was significantly lower (P=0.047) in the case group compared with the control group (OR, 0.611; 95% CI, 0.374–0.997), indicating that the T allele conferred a protective effect in CRC. The frequency of the AT genotype among the subjects diagnosed at >45 years of age was lower than those diagnosed at a younger age (P<0.05). The present study demonstrates that the T allele of the D1822V polymorphism may exert a protective effect against CRC, however, these findings require further validation in a larger sample size.

## Introduction

Colorectal cancer (CRC) is the third most common malignancy in the world and is one of the most frequent malignancies in China (1). In Shanghai, Beijing and other major cities, the increase in the incidence of CRC has been much faster compared with Western countries, and is associated with a corresponding rise in CRC mortality. This is the result of changes in living standards and diet coupled with the effect of genetic factors. Thus, the development of CRC is the result of an interaction between genetic, lifestyle and environmental factors.

**Figure 1:**
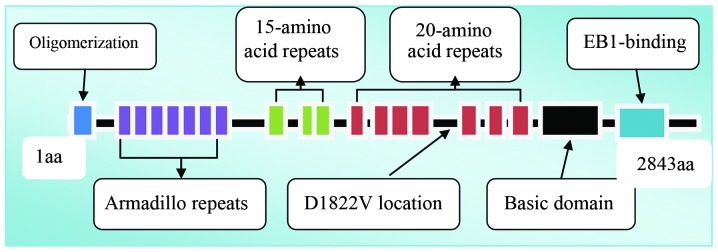
APC protein functional areas and APC gene D1822V single nucleotide polymorphism (SNP) of the encoded protein in APC. APC, adenomatous polyposis coli; aa, amino acid; EB1, end-binding protein 1.

Adenomatous polyposis coli (APC) has been identified as a tumor suppressor gene, located on chromosome 5q21-22, with a full-length of 11,025 bp. The cDNA sequence of APC is composed of 8,538 nucleotides that encode 2,843 amino acids (2). The APC protein has multiple functional areas, including an oligomerization and armadillo area in the N-terminal region, 15 and 20 AA nucleotide repeated sequences in the intermediate region and a conserved area that connects end-binding protein 1 and human disc-large protein in the C-terminal region (3) (Fig. 1).

Gene polymorphisms define the predisposition to human disease that arises from variations in genome sequences (4,5). Polymorphisms also account for different responses to medications and varied responses to environmental factors (6,7). The most common and simple form of gene polymorphisms are single nucleotide DNA sequence polymorphisms at the level of the genome.

Mutations in the APC gene have been shown to be responsible for the autosomal dominant inherited disease, familial adenomatous polyposis (FAP). The types of mutation include missense mutations, small insertions or deletions. These mutations result in the early appearance of the stop codon, with a subsequent generation of truncated APC protein (8), and aberrant cellular proliferation, leading to the early stages of CRC. Recent reviews and meta-analyses have indicated that APC is a candidate gene for the susceptibility to colorectal neoplasia (9). Codon 1,822 of the APC gene is identified by an A-to-T transversion, which causes a change in the amino acid sequence from aspartate to valine.

These findings indicate that it is possible that a single nucleotide polymorphism (SNP) may form the genetic basis for different individuals with FAP, with similar lifestyles to each other, having varied susceptibilities to CRC. D1822V is the most common APC variant described in the literature (10). However, no studies have investigated the association between this variant and the susceptibility of sporadic CRC in the Chinese mainland population. The present study was, therefore, undertaken to explore this correlation and to determine whether D1822V variants are associated with CRC in the population of Hubei, China.

## Material and methods

### Specimen collection

The present case-control study consisted of 196 patients with newly diagnosed and histopathologically confirmed primary CRC who had not previously undergone radiotherapy or chemotherapy. The patients were all admitted to the Zhong Nan Hospital of Wuhan University (Wuhan, Hubei, China) between February 2010 and November 2011. The control group included 279 histopathologically-confirmed cancer-free subjects with no current or previous diagnosis of cancer, who underwent health examinations at the Zhong Nan Hospital during the same period. The cases and controls were all residents of Hubei, and were matched for age and gender frequency using a uniform questionnaire, which included demographic characteristics, details of medical and family history and other information.

The study was approved by the Investigation and Ethics Committee of the Zhong Nan Hospital and was undertaken in accordance with the Declaration of Helsinki 1975. All subjects provided written informed consent prior to participating.

### Genomic DNA preparation

Venous blood (20 ml) was obtained in the morning from all the cases. Genomic DNA was isolated from whole blood using a Blood Genome DNA Extraction kit (Tiangen Biotech (Beijing) Co., Ltd., Beijing, China). Ultraviolet spectrophotometry was used to detect the content and concentration (A260/A280>1.8) of total DNA. Agarose gel electrophoresis (Biowest Agarose G-10; Gene Company Limited, Chi Wan, Hong Kong) was used to detect DNA integrity. The DNA samples were diluted to 100 ng/ml in sterile triple-distilled water and stored at 4°C.

### Polymerase chain reaction (PCR) and product determination

The upstream, 5′-ACCCAACAAAAATCAGTTAGATG-3′, and downstream, 5′-GTGGCTGGTAACTTTAGCCTC-3′, primer were provided by Invitrogen Life Technologies (Shanghai, China). PCR reactions were performed using a 20-μl reaction system containing 2 μl DNA template (100 ng/μl), 0.4 μl of each primer, 2 μl dNTPs, 0.2 μl ExTaq enzyme, 2 μl 10× PCR Buffer and 13 μl ddH_2_O. The PCR profile consisted of an initial 5-min denaturation step at 94°C, followed by 32 cycles of 30 sec at 94°C, 30 sec at 58°C and extension for 45 sec at 72°C, and a final 10-min elongation step at 72°C. The final PCR product was 410 bp.

Agarose gel (1%) electrophoresis (Extraction kit, Hangzhou Bioer Technology Co., Ltd., Hangzhou, Zhejiang, China) was used to recover the PCR products. The PCR fragments were sequenced at the Magic Biotech Company (Shanghai, China).

### Statistical analysis

Data analysis was performed using SPSS version 17.0 software (SPSS, Inc., Chicago, IL, USA). Bilateral χ^2^ tests were used to analyze demographic variables, environmental risk factors and APC genotype distributions between the case and control groups. Hardy-Weinberg equilibrium for genotypic frequencies was determined using the goodness-of-fit χ^2^ test. Univariate and multivariate unconditional logistic regression analyses were used to obtain odds ratios (OR) and corresponding 95% confidence intervals (CI). Stratified analysis was used to assess the effect of various factors on the incidence of CRC. P<0.05 was considered to indicate a statistically significant difference.

## Results

### Subject characteristics

**Figure 2:**
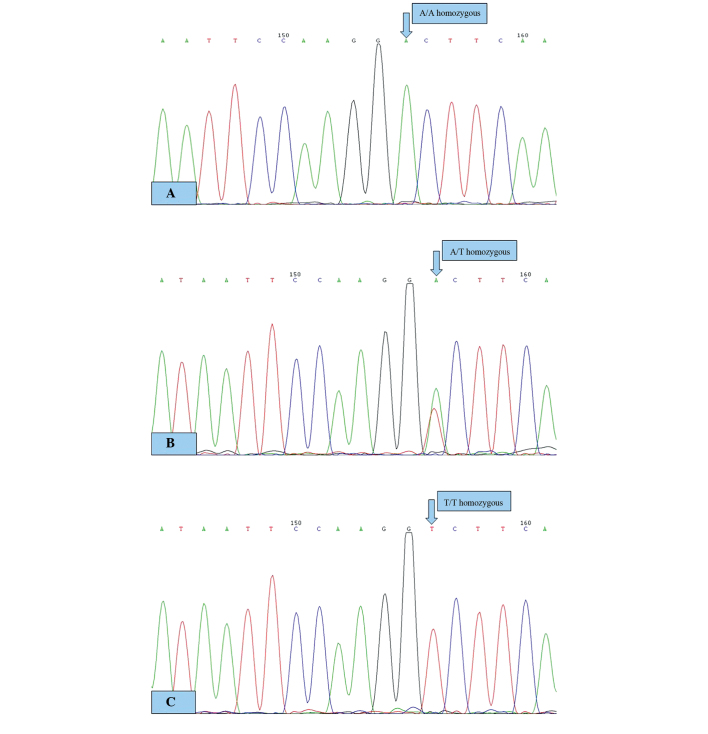
Sequencing peaks. The arrows are pointing to (A) a single peak representing the AA genotype; (B) a bimodal representative of the TA genotype; and (C) a single peak representing the TT genotype.

The direct sequencing histogram of the PCR products is shown in Fig. 2. A single peak represents homozygous products and double peaks represent heterozygous products.

The characteristics of the cases and controls are shown in Table I. The mean age of the case group was 59.6±12.9 years compared with 59.2±13.0 years in the control group (P=0.463). Similarly, there was no significant difference in gender distribution between the cases and controls (P=0.591).

### APC genotype and allele frequencies of the two groups

The APC gene AA genotype was present in 226 subjects in the normal control group, the AT genotype was found in 50 subjects and the TT genotype in three subjects. The T allele frequency was 0.1004 and the A allele frequency was 0.8996. The genotype distributions of APC D1822V were in agreement with the Hardy-Weinberg equilibrium (P=0.95).

In the case group, the AA genotype was present in 172 cases, the AT genotype in 23 cases and the AT genotype in one case. The T allele frequency was 0.0681 and the A allele frequency was 0.9362. The results are shown in Table II.

### Association between the APC gene D1822V polymorphism and the susceptibility to CRC

There were no significant differences between the case and control groups with respect to the ratio of the AT (OR, 0.604; 95% CI, 0.355–1.029) and TT (OR, 0.438; 95% CI, 0.045–4.247) genotypes compared with the AA genotype (Table II). The proportion of T alleles was significantly higher in the case group compared with the control group (P=0.047). The OR (95% CI) was 0.611 (0.374–0.997). These findings indicate that the T allele may be a protective factor for CRC.

### Associations between the APC gene D1822V polymorphism and the clinical features of CRC

There was no significant association between the APC D1822V SNP and the risk of CRC among subjects of a different gender or smoking and alcohol status. In addition, there was no link between the CRC risk and pre-existing hypertension or diabetes (Table III). However, the frequency of AT genotype was higher in the subjects that were >45 years old at the time of diagnosis compared with those <45 years old (P<0.05). The OR (95% CI) was 0.271 (0.093–0.790). This result indicates that the AT genotype may correlate with age to predict the risk of developing CRC. Thus, the AT genotype may play a protective role in the subgroup of patients >45 years old (Table III).

The effect of the D1822V SNP in APC was further evaluated to investigate possible associations with the clinicopathological characteristics of CRC. However, the variant homozygote TT genotype showed no significant association with the depth of tumor invasion, lymph node metastasis or the tumor location (Table III).

## Discussion

The development of CRC has been shown to result from an interaction of multiple factors. APC gene mutation is a cause of the inherited autosomal dominant disease, FAP. The types of mutation involved in this disease process include missense mutations, small insertions or deletions. Each of these has the potential to advance the stop codon, resulting in the generation of a truncated APC protein, with altered function (8). Mismatched repair gene mutations are associated with another rare disease known as hereditary non-polyposis CRC (11). However, these two hereditary diseases account for only 1–2% of all cases of CRC (11).

Associations between genotype and phenotype indicate that different types of APC mutations will have varying clinical manifestations (12). Although the presence of the D1822V SNP in the APC gene has been reported, the most frequently occurring mutation of this type, the prevalence of the AT genotype, has been reported to be as high as 30% in control populations (13).

Codon 1,822 is located between the 4th and 57th amino acid repeat region in the APC protein. The wild-type in this area has the effect of downregulating β-catenin (Fig. 1). The specific biological function of this missense mutation remains uncertain. However, it is believed to be a result in a change from aspartate to valine, which may have important clinical significance.

Recently, a review and meta-analysis indicated that APC is a candidate gene for colorectal neoplasia susceptibility, and provided evidence that APC mutation may result in aberrant cellular proliferation, leading to the early stages of CRC (7). Based on this assumption, the possible associations between the APC gene D1822V SNP and the susceptibility to CRC was investigated.

There are currently >20 ways to detect SNPs. These include TaqMan, mass spectrometry, chip methods and direct sequencing (14). Of these options, direct gene sequencing is the most reliable, and this was the method used in the present study. For cases of uncertain sequencing, fragments from the genome were re-amplified and subsequently the product was re-sequenced. This procedure provided a high level of accuracy.

The majority of studies on SNPs use peripheral blood, which is more convenient and more economical, particularly for patients with unresectable lesions. A number of studies have confirmed that oncogene mutations, consistent with the primary tumors, are present in DNA extracted from peripheral blood (15–17). Based on this experience, the present study used direct sequencing to genotype peripheral blood specimens collected from 196 CRC cases and 279 healthy controls. The frequency of the T allele in the control group was similar to that reported in the in HapMap Project of the Chinese Han population in Beijing (18). The frequency of the AT and TT genotypes was significantly lower than the frequency of the AA genotype in the case and control groups. However, the frequency of the A allele was significantly higher compared with the T allele in the groups (P=0.047), indicating that the T allele may be a protective factor for CRC. These results are in accordance with previous findings showing that AT heterozygotes for this site were associated with a low odds ratio for CRC, indicating that the presence of the T allele at this site may protect against the development of CRC (8). However, no TT homozygotes were identified in this previous population.

In another study, APC D1822V was shown to exert a protective effect in patients with colon (OR, 0.76; 95% CI, 0.60–0.97) and rectal cancer (OR, 0.73; 95% CI, 0.56–0.95), respectively (18). A study using the denaturing high-performance liquid chromatography technique also showed that individuals with homozygous mutant alleles of the variant were at a reduced risk of developing CRC (19). However, a study in Tunisian subjects found no statistically significant association between the D1822V variant and CRC risk (20).

APC D1822V is traditionally considered to be a risk factor for colorectal tumorigenesis. In 1999 (21), a VV homozygous mutation was found in patients with FAP, and also in a proportion of healthy subjects. However, APC D1822V was not found in the control population, indicating that the D1822V mutation may be a morbid change that is associated with an increased risk of developing CRC.

An increasing body of research has focused on investigating the association between the APC D1822V variant and CRC risk. In a previous study, the D1822V variant of the APC gene was found not to be associated with a family history of CRC, but it has the potential to increase the risk of adenoma transformation (22). In the same study, individuals with the AA genotype who had a history of symptomatic adenomas had a 2-fold increase in the risk of developing CRC (OR, 1.91; 95% CI, 0.56–6.52; P=0.02). This risk further increased in T allele carriers.

Another large study (23) found an association between the TT genotype and a reduced risk of colon cancer among individuals diagnosed at >65 years of age, and among those maintained on a low-fat diet. This finding is partly consistent with a study demonstrating a protective role of the TT genotype in CRC, in the absence of any consistent association between the protective effect of D1822V specific to patients with a low-fat diet or advanced age (13). A further study in 196 cases and 200 controls was undertaken to explore the interaction between the D1822V polymorphism, APC gene and dietary intake in subjects with CRC (24). A study that evaluated nutritional status and lifestyle found that a high cholesterol intake was associated with an increased risk of CRC (OR, 1.66; 95% CI, 1.00–2.76) only in non-carriers (DD) of the D1822V APC allele. A large case-control study in Scotland also reported a reduced risk of CRC in individuals consuming a diet that was low in fat (total, trans and saturated and monounsaturated fatty acids) (25). However, this study did not observe any associations between these variants and the CRC risk in the population as a whole.

Each of these previously reported studies supports the findings of the present study, showing that the T allele for the APC gene D1822V variant appears to confer a protective effect against CRC. Other studies have shown that the APC Asp1822Val and Gly2502Ser polymorphisms are not associated with an increased risk of CRC or adenoma (26). However, the same studies found a significant correlation between the Asp1822Val genotype and the use of a postmenopausal hormone (P=0.03). All these results support the idea that the incidence of CRC is due to genetic, environmental and lifestyle factors.

The present study lacks information on diet, and was therefore unable to analyze the interaction between diet, lifestyle and genetic factors. The study population was also relatively small, and larger studies will therefore be required to validate its findings. The present study does, however, provide evidence for the first time that the T allele of the APC gene D1822V polymorphism may be a protective factor for CRC in Chinese subjects, particularly in those >45 years of age.

## Figures and Tables

**Table I:** Demographic characteristics of the cases (n=196) and controls (n=279).

Characteristics	Case, n (%)	Controls, n (%)	χ^2^	P-value
Gender				
Male	126 (64.29)	185 (66.31)	0.290	0.591
Female	70 (35.71)	94 (33.69)		
Age, years				
<65	122 (62.24)	188 (67.38)	1.341	0.247
≥65	74 (37.76)	91 (32.62)		

**Table II:** APC gene D1822V SNP and colorectal cancer risk.

Genotype	Cases, n	Controls, n	OR (95% CI)	P-value
A/A	172	226	1.00	
A/T	23	50	0.604 (0.355–1.029)	0.062
T/T	1	3	0.438 (0.045–4.247)	0.822
A	367	502	1.00	
T	25	56	0.611 (0.374–0.997)	0.047

APC, adenomatous polyposis coli; OR, odds ratio; CI, confidence interval; SNP, single nucleotide polymorphism.

**Table III:** Stratified analyses for the variant D1822V genotype in the cases and controls.

Variable	AT	AA	OR (95% CI)	P-value
Gender	23	172		
Female	8	62	1.00	
Male	15	110	1.057 (0.424–2.633)	0.906
Age, years				
<45	6	15	1^a^	
≥45	17	157	0.271 (0.093–0.790)	0.012
Smoking status				
Non-smokers	13	115	1.00	
Smokers	10	56	1.580 (0.653–3.824)	0.308
Alcohol consumption				
No	18	139	1.00	
Yes	5	32	1.207 (0.417–3.492)	0.729
Hypertension				
Yes	18	132	1.00	
No	5	39	0.940 (0.328–2.695)	0.909
Diabetes				
No	20	154	1.00	
Yes	3	17	1.359 (0.366–5.050)	0.646
Depth of tumor invasion^b^,^c^				
Tis	0	6		
T1	1	3		
T2	4	20	−0.402	0.688
T3	6	65		
T4	12	74		
Lymph node metastasis^a^				
Positive	8	80	1.00	
Negative	15	91	0.607 (0.244–1.506)	0.278
Location				
Colon	12	77	1.00	
Rectum	11	95	0.743 (0.311–1.776)	0.503

a Lymph node status of one patient could not be determined;

b pathological data could not be determined for four patients;

c Mann-Whitney U test.

OR, odds ratio; CI, confidence interval.
